# Postictal aggression in epilepsy: prevalence, clinical correlates, and psychosocial impact

**DOI:** 10.1007/s00415-026-13796-z

**Published:** 2026-04-07

**Authors:** Isabelle Herion, David Steinbart, Desislava Dimova, Martin Holtkamp

**Affiliations:** 1 Department of Neurology, Charité - Universitätsmedizin Berlin, corporate member of Freie Universität and Humboldt-Universität Zu Berlin, Augustenburger Platz 1, 13353 Berlin, Germany; 2 Department of Neurology, University Hospital Augsburg, Stenglinstr. 2, 86156 Augsburg, Germany; 3 Epilepsy-Center Berlin-Brandenburg, Institute for Diagnostics of Epilepsy, Herzbergstr. 79, 10365 Berlin, Germany

**Keywords:** Antiseizure medication, Anxiety, Postictal agitation, Quality of life

## Abstract

**Objective:**

Postictal aggression (PIA) is an under-researched phenomenon with potentially harmful consequences for patients and their environment. This study assessed the prevalence of PIA, its associated clinical variables, and its relationship with quality of life (QoL), anxiety, and depression.

**Methods:**

In this cross-sectional study, consecutive patients with epilepsy (≥ 1 seizure in the past 12 months; age ≥ 16 years) were surveyed using standardized questionnaires in a tertiary epilepsy center. Occurrence of PIA was retrospectively assessed over the preceding 12 months and defined by a score of ≥ 2 on the Overt aggression scale modified (OAS-M). Anxiety and depression were measured with the Hospital anxiety and depression scale (HADS-A, HADS-D) and the Neurological disorders depression inventory in epilepsy (NDDI-E).

**Results:**

A total of 201 patients were included (57% male; median age 47 years; 76% focal epilepsy, 14% idiopathic generalized epilepsy (IGE), 10% unclassified). Twenty-four patients (12%) reported clinically relevant PIA. Patients with PIA had lower QoL (QOLIE-10 median 40 vs. 37, p = 0.031) and more frequent clinically relevant anxiety (HADS-A ≥ 8: 58% vs. 30%, p = 0.019). Depressive symptoms were more common in PIA (NDDI-E ≥ 16: 21% vs. 10%) without statistical significance (p = 0.309). IGE (OR 3.13, 95% CI 1.06–8.98) and anxiety (OR 2.51, 95% CI 1.02–6.26) were independently associated with PIA. There were no associations with antiseizure medications or their dosage. Ten patients reported adverse consequences of PIA, most commonly shame and fear.

**Conclusion:**

Almost one in eight patients experienced PIA. It was associated with reduced QoL and increased anxiety, highlighting the psychosocial burden.

**Supplementary Information:**

The online version contains supplementary material available at 10.1007/s00415-026-13796-z.

## Introduction

The postictal state can manifest with a huge variability of neurological and behavioral signs [1]. Postictal aggression (PIA) poses a particularly challenging phenomenon, characterized by spontaneous, directed physical or verbal hostility of brief duration, emerging within minutes to hours after a seizure and lasting from several minutes up to several days [2–4]. Affected individuals typically express full remorse following such episodes [2, 3] and characteristically do not experience psychotic episodes or personality change interictally [2]. Differentiating PIA from postictal confusion and postictal psychosis is essential. In postictal confusion, violent behavior appears almost immediately after the seizure and is generally undirected and resistive, often triggered when the patient is physically restrained [2]. In contrast, postictal psychosis may include directed aggressive actions as a secondary consequence of psychotic experiences as persecutory delusions, threatening hallucinations and mood disturbances, but typically occurs after a lucid interval of few hours to days after a cluster of seizures [5–7].

Reported prevalence rates of PIA vary widely across studies, ranging from 0.5% of patients in a cohort of unselected 1300 individuals [3] and 1.2% in 170 video-EEG-recorded seizures in a study of 20 patients evaluated in an epilepsy monitoring unit [8] to a prevalence of 52% among 126 patients treated in a specialized tertiary epilepsy center [4]. Several case reports also describe episodes of PIA [2, 9–11]. The occurrence of PIA has been associated with a longer duration of epilepsy and the use of a higher number of antiseizure medications (ASM) [4]. Although severe individual negative consequences of PIA have been well described [2, 3], a comprehensive analysis of the prevalence together with its psychosocial impact, particularly on quality of life, has not yet been conducted. In general, comparability across studies is limited by heterogeneous or vaguely specified definitions of PIA, including inconsistent differentiation from general postictal confusion with violent behavior or postictal psychosis. So far, no standardized operational definition of PIA has been established.

Considering its potential for significant individual harm and absence of systematic data, a clearer delineation of PIA is needed. Therefore, this study evaluated the prevalence of PIA based on the Overt aggression scale modified (OAS-M) in a cohort of consecutive patients with epilepsy and assessed independently associated variables, its impact on quality of life (QoL) and potential associations with anxiety and depression.

## Materials and methods

### Patient selection

Consecutive patients with epilepsy were recruited between April 2021 and February 2022 from the three epilepsy outpatient clinics affiliated with the Department of Neurology at the Charité—Universitätsmedizin Berlin and from the outpatient clinic at the Epilepsy-Center Berlin-Brandenburg at the Evangelisches Krankenhaus Königin Elisabeth Herzberge, Berlin, Germany. Patients had to have at least one unprovoked seizure within the last 12 months to be included. Patients with only functional/dissociative seizures or only acute symptomatic seizures were excluded. Further, patients with moderate to severe intellectual disability, legal guardianship and patients with insufficient German language skills were excluded. Also, patients and their proxies were inquired about postictal delusions, misperceptions, persecutory ideation, and misidentification of familiar persons. Patients with any of those postictal psychotic signs or symptoms were excluded to clearly distinguish postictal aggression from postictal psychosis.

This study was carried out in accordance with the World Medical Association Declaration of Helsinki and it was approved by the Institutional Review Board of the Charité—Universitätsmedizin Berlin (EA2/306/20). All participants received written study information prior to inclusion and signed informed consent forms. Data were handled in compliance with German and European data protection regulations.

### Outcome measures and data acquisition

Basic demographic and clinical data were obtained from electronic databases. ASM dosages were registered as defined daily dose (DDD) ratios (individual ASM dosage divided by DDD) based on the ATC/DDD Index of the WHO Collaborating Centre for Drug Statistics Methodology [12]. To assess signs of postictal aggression within the preceding 12 months, patients were cross-sectionally interviewed using a standardized questionnaire (Overt aggression scale-modified, OAS-M) [13]. The OAS-M is a widely used instrument for evaluating aggressive behavior covering the following four domains: verbal aggression, aggression against objects, aggression against other individuals and aggression against self (Table S1) [14]. The presence of postictal aggression was operationally defined as an OAS-M score of ≥ 2. In addition, patients were asked prior to the OAS-M interview if they had noticed any subjective hints of postictal and interictal aggression. If possible, an additional interview was conducted with a family member or a close relative to acquire an external assessment of postictal and interictal aggression.

Subsequently, the participants completed a survey including the following five questionnaires:

1. **Liverpool seizure severity scale** (LSSS; 20 items; best score 20, worst score 80) [15], which evaluates frequency, duration and semiology of seizures, seizure-related injuries and postictal state.
2. **Hospital anxiety and depression scale** (HADS; consisting of two subscales: Anxiety (HADS-A) and Depression (HADS-D), each containing seven intermingled items, each maximum score 21) [16, 17], capturing core symptoms of anxiety and depression over the past week, while largely excluding somatic manifestations. A cutoff score of ≥ 8 is considered indicative for anxiety [17] and depression [17, 18] respectively.
3. **Neurologic disorders depression inventory for epilepsy** (NDDI-E; 6 items, maximum score 46) [19], which comprises items on guilt, self-esteem, frustration, anhedonia, and suicidal thoughts to reflect the past 2 weeks. A cutoff score of ≥ 16 is considered indicative for depression [18, 19].
4. **Quality of life inventory** (QOLIE-10-P; best score 100, worst score 0) [20], with Part A assessing impairments in daily life, medication effects, and seizure-related fears, while Part B addresses overall quality of life.
5. **Liverpool adverse events profile** (LAEP; 19 items; best score 19, worst score 76) [21, 22], which measures common cognitive, dermatological, gastrointestinal, neurological and psychological adverse events associated with ASM.

### Data analysis

Continuous variables are displayed as median and interquartile ranges (IQR), and non-parametric tests were used due to skewed data distributions. Categorical variables were compared using Chi-squared or Fisher’s exact tests, as appropriate. The Benjamini–Hochberg method was applied to correct for multiple testing and adjusted p-values are reported as appropriate.

Least absolute shrinkage and selection operator (LASSO) logistic regression with tenfold cross-validation was used to select relevant variables for postictal aggression. Candidate predictors included age at interview, age at epilepsy onset, sex, epilepsy type, number of seizures in the last 12 months, total ASM drug load (registered as DDD ratios), current use of levetiracetam, brivaracetam or perampanel, seizure severity (LSSS), and clinically relevant symptoms of anxiety (HADS-A ≥ 8) as well as depression (HADS-D ≥ 8). Variables retained in the LASSO model were subsequently entered into a Firth logistic regression.

The significance threshold was set at p = 0.05.

All analyses were conducted using IBM SPSS Statistics 30.0 (IBM Corp, Armonk, NY, U.S.A.) and R version 4.4.2 (http://www.r-project.org).

## Results

### Prevalence and characteristics of participants with postictal aggression

In the study period, 208 individuals were initially enrolled, of which 7 participants had to be excluded (3 participants without epilepsy, 3 participants with postictal psychotic signs, 1 participant due to insufficient documentation). Eventually, 201 patients were included and available for further analysis. Demographical and clinical characteristics of the cohort can be found in Table 1.

**Table 1:** Demographic and clinical data

Item		All patients n = 201	Patients with PIA (OAS-M ≥ 2) n = 24	Patients without PIA (OAS-M < 2) n = 177	Test statistics
Sex n (%)	Male	114 (56.7%)	9 (37.5%)	105 (59.3%)	x^2^ = 4.10 p_raw_ = 0.043 **p**_**adj**_** = 0.043** φ = 0.14
	Female	87 (43.3%)	15 (62.5%)	72 (40.7%)	
Age at interview (years)	Median (IQR)	47 (31–62)	38 (24–57)	49 (32–64)	W = 1504 p_raw_ = 0.020 **p**_**adj**_** = 0.043** r = −0.16
Age at onset of epilepsy (years)	Median (IQR)	22 (14–40)	16 (12–23)	24 (14–40)	W = 1550 p_raw_ = 0.032 **p**_**adj**_** = 0.043** r = −0.15
Epilepsy type n (%)	Focal	153 (76.1%)	14 (58.3%)	139 (78.5%)	x^2^ = 4.74 p_raw_ = 0.029 **p**_**adj**_** = 0.044** φ = 0.154
	Idiopathic generalized	29 (14.4%)	8 (33.3%)	21 (11.9%)	Fisher’s OR = 3.68 p_raw_ = 0.010 **p**_**adj**_** = 0.031**
	Unclassified	19 (9.5%)	2 (8.3%)	17 (9.6%)	Fisher’s OR = 0.86 p_raw_ = 1
Seizure types	Number of focal preserved consciousness seizures (in last 12 months) Median (IQR)	24 (6–88) (n = 75, 37%)	48 (2–103) (n = 12, 50%)	24 (6–88) (n = 63, 36%)	W = 386 p_raw_ = 0.914 p_adj_ = 0.914 r = 0.01
	Number of focal impaired consciousness seizures (in last 12 months) Median (IQR)	9 (4–50) (n = 73, 36%)	23 (2–136) (n = 10, 42%)	9 (4–49) (n = 63, 36%)	W = 328 p_raw_ = 0.841 p_adj_ = 0.914 r = 0.02
	Number of bilateral tonic–clonic seizures/generalized tonic–clonic seizures (in last 12 months) Median (IQR)	2 (1–3) (n = 108, 54%)	3 (3–7) (n = 8, 33%)	2 (1–2) (n = 100, 57%)	W = 695 p_raw_ = 0.011 **p**_**adj**_** = 0.044** r = 0.24
	Number of typical absence seizures (in last 12 months) Median (IQR)	66 (53–166) (n = 8, 4%)	66 (58–253) (n = 3, 13%)	65 (53–75) (n = 5, 3%)	W = 7 p_raw_ = 1 p_adj_ = 1 r = 0
Liverpool seizure severity scale	Median (IQR)	49 (43–55)	49 (45–52)	49 (43–55)	W = 2088 p_raw_ = 0.852 p_adj_ = 0.914 r = 0
Antiseizure medication	Number of current ASM Median (IQR)	1 (1–2)	1 (1–2)	1 (1–2)	W = 2264 p_raw_ = 0.556 p_adj_ = 0.556 r = 0
	Number of all ASM (current and history) Median (IQR)	3 (1–4)	4 (2–4)	3 (1–4)	W = 2497 p_raw_ = 0.157 p_adj_ = 0.315 r = 0.10
	ASM total drug load ratio (individual ASM dosage divided by DDD) Median (IQR)	1.5 (1.0–2.6)	1.6 (1.2–2.5)	1.5 (0.8–2.6)	W = 2369 p_raw_ = 0.359 p_adj_ = 0.479 r = 0.06
Liverpool adverse events profile	Median (IQR)	37 (28–45)	42 (35–48)	36 (28–45)	W = 2651 p_raw_ = 0.021 p_adj_ = 0.084 r = 0.17
Subjective interictal general aggressiveness or irritability n, (%)		23 (11.3%)	6 (25%)	17 (9.4%)	Fisher OR = 0.30 p_raw_ = 0.031 **p**_**adj**_** = 0.031**

Comparison of demographic and clinical data between patients with postictal aggression (PIA), defined by Overt aggression scale modified (OAS-M) ≥ 2, and patients without PIA

*ASM* antiseizure medication, *DDD* defined daily dose; *IQR* interquartile range; *n* number of patients; *OAS-M* overt aggression scale modified; p-values were adjusted for multiple comparison following Benjamini–Hochberg method (within contextually related blocks); p-value was significant if < 0.05; significant p-values are highlighted in bold

Twenty-four participants (12%) fulfilled the criterium for PIA following the OAS-M (score ≥ 2), of which 23 participants (96%) prior to OAS-M assessment self-reported subjective occurrence of PIA. An additional number of 10 patients self-reported experience of PIA without meeting formal definition (with OAS-M scores < 2) (Table S2).

External proxy reports were available for 30 patients. Five proxies (17%) reported signs of PIA as defined by an OAS-M score ≥ 2, and these accordant five proxies had already reported a subjective occurrence of PIA prior to the structured OAS-M interview (Table S2).

A significantly higher portion of female patients (63 vs. 41%, p = 0.043) and a younger age at epilepsy onset (median 16 vs. 24 years, p = 0.043) were observed in participants with PIA (n = 24) compared to those without (n = 177). One third of the 24 participants with PIA had IGE (12% of patients without PIA, p = 0.031), 58% had focal epilepsy (79% of patients without PIA, p = 0.044). PIA was most frequently self-reported after bilateral tonic–clonic/generalized tonic–clonic seizures (BTCS/GTCS; n = 14), followed by focal impaired awareness seizures (n = 11), focal aware seizures (n = 10), and less frequently in patients with typical absence seizures (n = 2), with multiple seizure types possibly occurring in the same patient (Table S3).

The overall number of BTCS/GTCS in the previous 12 months was higher in patients with PIA (median 3 vs. 2, p = 0.044), without any significant differences in number of other seizure types. There were no significant differences in general seizure severity (measured by LSS), number and cumulative dosage of ASM as well as adverse events as measured by LAEP (Table 1). Lamotrigine was the most frequent ASM administered to patients with PIA (50%), followed by levetiracetam (33%) and lacosamide (17%). There were no significant differences in the choice or dosage of ASM between patients with and without PIA (Table 2).

**Table 2:** Frequency of postictal aggression stratified by antiseizure medication

Antiseizure medication (n (%))	Patients with PIA (OAS-M ≥ 2) n = 24	Patients without PIA (OAS-M < 2) n = 177	Test statistics
Lamotrigine n (%)	12 (50.0%)	59 (33.3%)	x^2^ = 2.57 p = 0.545
Dosage mg Median (IQR)	388 (288–463)	400 (275–475)	W = 350 p = 0.945
Levetiracetam n (%)	8 (33.3%)	77 (43.5%)	x^2^ = 0.09 p = 0.573
Dosage mg Median (IQR)	2,625 (1,375–3,125)	2,000 (1,000–3,000)	W = 372 p = 0.329
Lacosamide n (%)	4 (16.7%)	38 (21.5%)	x^2^ = 0.29 p = 0.696
Dosage mg Median (IQR)	350 (275–425)	325 (200–438)	W = 80 p = 0.879
Valproate n (%)	4 (16.7%)	14 (7.9%)	Fisher OR = 2.32 p = 0.573
Dosage mg Median (IQR)	1,500 (1,200–1,850)	1,650 (1,050–2,000)	W = 28 p = 0.957
Perampanel n (%)	2 (8.3%)	13 (7.3%)	Fisher OR = 1.11 p = 0.696
Dosage mg Median (IQR)	10 (9–11)	6 (6–8)	W = 22 p = 0.136

Most frequently administered antiseizure medications (ASM) in mono- or polytherapy, stratified by occurrence of postictal aggression, defined by Overt aggression scale modified (OAS-M) ≥ 2. Less frequently administered ASM are listed in Table S4. n = number of patients; IQR: interquartile range

### Comorbid anxiety and depression

**Fig. 1:**
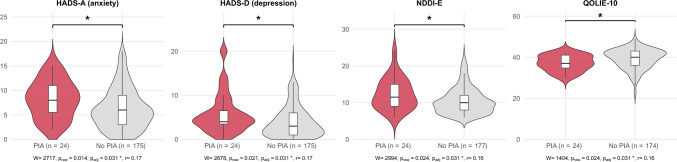
Comparison of psychiatric comorbidities, measured by Hospital anxiety and depression scale (HADS-A and HADS-D) with respective subscales and Neurological disorders depression inventory in epilepsy (NDDI-E), and quality of life measured by Quality of life in epilepsy questionnaire (QOLIE-10) between patients with postictal aggression (PIA, defined by Overt aggression scale modified ≥ 2) and those without. *N* number of patients

**Fig. 2:**
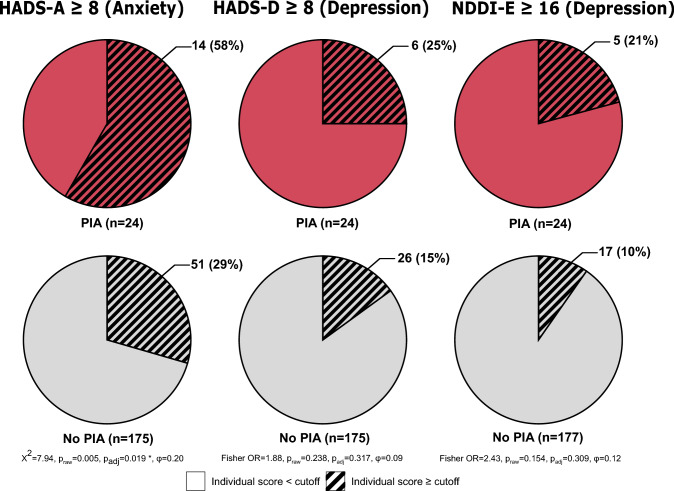
Proportion of patients exceeding cutoff scores on the Hospital anxiety and depression scale (HADS) subscales and on the Neurological disorders depression inventory in epilepsy (NDDI-E). Scores above cutoff were considered indicative of anxiety (HADS-A) or depression (HADS-D, NDDI-E). Proportions are shown separately for patients with postictal aggression (PIA; Overt aggression scale modified ≥ 2) and those without. *N* number of patients

In the group of patients with PIA, median HADS-A scores were significantly higher compared to patients without PIA (median 8 (IQR 6–11) vs. median 6 (IQR 3–8), p = 0.031, Fig. 1). A significantly larger proportion of patients with PIA also reached a score of ≥ 8, indicating clinically relevant anxiety symptoms (58% vs. 29%, p = 0.019, Fig. 2).

Patients with PIA showed significantly higher depressive symptom scores on both the HADS-D (median 4 (IQR 4–6) vs. median 3 (IQR 1–6), p = 0.031) and the NDDI-E (median 4 (IQR 4–6) vs. median 3 (IQR 1–6), p = 0.031, Fig. 1). Although the proportion of patients meeting criteria for clinically relevant depressive symptoms was higher in the PIA group for both measures, these differences did not reach statistical significance (Fig. 2).

### Factors independently associated with postictal aggression

In the logistic regression analysis (LASSO selection followed by Firth logistic regression), IGE (OR 3.13, 95% CI 1.06–8.98) and clinically relevant anxiety symptoms (OR 2.51, 95% CI 1.02–6.26) were independently associated with the occurrence of PIA. The number of focal impaired consciousness seizures in the previous 12 months was also statistically associated with PIA, although with a very small effect size (OR 1.01, 95% CI 1.00–1.01). In the multivariable model, the number of BTCS/GTCS in the previous 12 months was not independently associated with PIA (Table 3).

**Table 3:** Variables independently associated with postictal aggression (OAS-M ≥ 2)

Variable	Odds ratio (95% CI)	p-value
Sex (female)	2.30 (0.94–5.94)	0.070
Age at interview (per year)	0.98 (0.95–1.01)	0.118
Age at first seizure (per year)	not selected by LASSO	
Epilepsy type (idiopathic generalized vs. focal)	**3.13 (1.06–8.98)**	**0.039**
Epilepsy type (unclassified vs. focal)	1.33 (0.23–5.54)	0.723
Number of BTCS/GTCS (in the last 12 months)	not selected by LASSO	
Number of FPCS (in the last 12 months)	not selected by LASSO	
Number of FICS (in the last 12 months)	**1.01 (1.00–1.01)**	**0.039**
ASM total drug load ratio (individual ASM dosage divided by DDD)	not selected by LASSO	
ASM: Levetiracetam	not selected by LASSO	
ASM: Brivaracetam	not selected by LASSO	
ASM: Perampanel	not selected by LASSO	
Liverpool seizure severity scale (per point)	not selected by LASSO	
Clinically relevant anxiety symptoms (HADS-A ≥ 8)	**2.51 (1.02–6.26)**	**0.045**
Clinically relevant depression symptoms (HADS-D ≥ 8)	not selected by LASSO	

Results of multivariable analysis on factors independently associated with postictal aggression (defined by Overt aggression scale modified (OAS-M) ≥ 2). Variable selection was performed using LASSO (Least Absolute Shrinkage and Selection Operator) logistic regression with tenfold cross-validation (λmin criterion). Selected predictors were re-estimated using Firth’s bias-reduced logistic regression on complete cases

N = 201; complete cases for LASSO/Firth = 191; AUC = 0.765 (95% CI 0.663–0.868); Nagelkerke’s R^2^ = 0.204

95% *CI* 95% confidence interval; *ASM* antiseizure medication; *BTCS/GTCS* bilateral tonic–clonic/generalized tonic–clonic seizures; *DDD* defined daily dose; *FPCS* focal preserved consciousness seizures; *FICS* focal impaired consciousness seizures; *HADS* Hospital anxiety and depression scale, consisting of the two subscales anxiety (HADS-A) and depression (HADS-D); *OAS-M* Overt aggression scale modified

### Quality of life and adverse consequences due to postictal aggression

Quality of life was lower in patients with PIA compared to those without, as reflected by lower QOLIE-10 scores (median 37 (IQR 35–41) vs. median 40 (IQR 36–43), p = 0.031; Fig. 1). Overall, 14 out of 24 individuals with PIA reported experiencing adverse consequences related to these episodes, most commonly feelings of shame (Table 4).

**Table 4:** Adverse consequences due to postictal aggression

Variable	Frequency in patients with PIA (OAS-M ≥ 2) n = 24
Any negative consequence (n(%))	14 (58.3%)
Shame	8 (57.1%)
Fear	4 (28.6%)
Mentally stressed	2 (14.3%)
Disputes with other people	2 (11.8%)

Frequency of self-reported adverse consequences due to postictal aggression (PIA), defined by Overt aggression scale modified (OAS-M) ≥ 2, detail percentages based on the group of patients with any negative consequence (n = 14); n: number of patients

## Discussion

In the current study, we comprehensively investigated the prevalence of PIA and its associations with clinical and demographic characteristics. In our cohort of 201 PWE, we found a prevalence of 12%. Due to heterogeneous methodological approaches, both in the definition of PIA and in the selection of study participants, comparability with previous studies is limited. The observed prevalence of PIA is markedly higher than the rates of 0.5% and 1.2% reported in two earlier studies with varying sample sizes [3, 8], yet substantially lower than the 52% reported in a cohort drawn from an epilepsy monitoring unit with a presumably different selection of patients and a different assessment of PIA [4]. We consider our findings to be based on a representative sample, as we included consecutive patients treated across multiple outpatient clinics in different regions of Berlin.

A clear definition of PIA is crucial for reliable investigation of its prevalence and associated factors, yet no consensus-based definition currently exists, and no scales or questionnaires have been specifically developed to assess postictal aggression. We based the operational definition of PIA on the OAS-M and deduced a cutoff score ≥ 2 from the validation study of Coccaro [14], in which individuals with intermittent explosive disorder were compared to healthy controls. The OAS-M total score is weighted, with aggression toward objects, others, or oneself multiplied by a factor of 2 (property aggression) or 3 (physical aggression) [14]. Thus, a total score of 2 reflects either clinically meaningful verbal aggression or any instance of physical aggression. On this basis, we considered an OAS-M score ≥ 2 an appropriate threshold for defining PIA. The Social dysfunction and aggression scale (SADS) [23] is an alternative tool to measure aggressive behavior, consisting of nine items. It was used in a previous study to quantify aggressive behavior in postictal psychosis [7]. As the OAS-M offers a more specific measure for aggressive behavior, we decided to use the OAS-M instead of the SADS as a suitable approximation for systematically capturing signs of PIA.

As data on prevalence of PIA have been limited, investigations into its underlying pathophysiology are likewise rudimentary. The independent association between PIA and IGE as observed in the current study may suggest that certain epileptogenic networks of generalized epilepsies facilitate the emergence of PIA. Based on current understanding, these IGE networks are characterized by reciprocal interactions between thalamic nuclei and widespread cortical and subcortical regions [24]. Moreover, abnormalities in the insula, anterior cingulate cortex, and basal ganglia – regions involved in aggression and affective processing – have been described in IGE [25, 26]. However, direct clinical associations between aggression and these network abnormalities have not been studied extensively. The pathogenesis of PIA likely reflects an interplay of different factors [1]. Malfunction of specific brain regions may arise through various postictal processes, including neurotransmitter depletion [27], increased extracellular potassium [27], postictal hypoperfusion [28] and hypoxia [29], disruption of the blood–brain barrier [30], and dysfunction of inhibitory interneurons [31, 32]. These neurobiological alterations presumably interact with individual vulnerabilities, including potential genetic predispositions [33], to shape the emergence and severity of PIA. The association between PIA and IGE suggests that PIA is unlikely to be mediated by structural brain lesions. Future studies integrating functional neuroimaging and tailored neuropsychological assessments may help to further elucidate potential shared underlying pathophysiological mechanisms.

The occurrence of PIA was independently associated with a higher rate of clinically relevant symptoms of anxiety, possibly reflecting a bidirectional relationship, as experiencing PIA may increase anxiety, while pre-existing anxiety may influence the perception and reporting of postictal behavioral changes. Furthermore, PIA was associated with reduced quality of life in our study. This finding corroborates results of a previous study that described a correlation between extent and intensity of constraints during the postictal state and subjective seizure severity as well as quality of life [34]. This result further supports the recommendation that treating physicians should proactively inquire about signs of PIA and systematically integrate this information into individualized treatment plans. Therapeutic strategies for managing PIA involve both non-medical and medical approaches. First, ensuring the safety of the patient and their surrounding by removing dangerous objects or closing windows and guiding the patient is paramount [35], although this can be challenging given the sudden onset and directed nature of the aggressive behavior. In cases of severe aggression, benzodiazepines (e.g., lorazepam or midazolam) or antipsychotic drugs (e.g., haloperidol or quetiapine) could be administered rapidly after the seizure as a symptomatic treatment [35]. As a reduction of seizure frequency is the most effective way to prevent postictal complications – as illustrated by the association between number of focal impaired consciousness seizures and PIA in the current study – an optimal seizure control is crucial. In cases of drug-resistant epilepsy, further interventions such as ablative or resective epilepsy surgery [36] or neurostimulation [37] should be considered as potential options to improve overall seizure control and thereby mitigate the risk of PIA.

Our study has strengths and limitations. The registration of PIA was based on a structured interview capturing self-reported PIA signs, aiming to achieve a valid assessment of PIA rather than solely assessing the subjective occurrence of PIA. Although the OAS-M cutoff score of ≥ 2 for defining PIA was derived from a previous study [14], this operational definition of PIA has not been formally validated before. Despite this, PIA defined by an OAS-M score ≥ 2 showed high concordance with subjective patient reports (96%) and with all available proxy reports, supporting the clinical plausibility of this OAS-M- based operationalization. External reports of family members or relatives were available for 15% of patients, which limited the possibility of systematically validating self-reported symptoms. However, in this study, patients’ self-reports of PIA were often based not only on their own recollection but also on accounts previously communicated to them by witnesses of the episodes, thereby indirectly reflecting external observations in many reports despite the absence of formal proxy interviews. Furthermore, the strict differentiation from postictal psychosis, achieved by excluding patients with any psychotic signs, facilitated a reliable characterization of PIA. Despite recruitment across four outpatient clinics in different regions of Berlin, the study was monocentric, potentially limiting generalizability. To maximize accessibility and ensure a high participation rate, PIA signs were assessed retrospectively. This approach may have constrained the accuracy of patients’ reporting. Furthermore, modifications of ASM intake prior to study inclusion could not be recorded systematically. Future studies could focus on a multicentric prospective registration of PIA signs to strengthen the data of PIA prevalence, further examine potential interactions with ASM, and investigate underlying pathophysiological mechanisms.

## Conclusion

Postictal aggression affected almost one in eight patients with epilepsy and was associated with a substantial individual burden, reflected by a higher prevalence of clinically relevant anxiety symptoms, reduced quality of life and a feeling of shame. When treating patients with epilepsy, PIA should be considered and specifically inquired about.

## Supplementary Information

Below is the link to the electronic supplementary material.

Supplementary file1 (DOCX 37 KB)

## Data Availability

Data available on reasonable request due to privacy and ethical restrictions.
