# Supplementary material for: Postictal aggression in epilepsy: prevalence, clinical correlates, and psychosocial impact
Source: J Neurol. 2026 Apr 7;273(5):254. doi: 10.1007/s00415-026-13796-z (PMC13056792; doi:10.1007/s00415-026-13796-z)
Supplement: Supplementary file 1 — Supplementary file1 (DOCX 37 KB) [file 415_2026_13796_MOESM1_ESM.docx]

**Supplementary Information for**

**Postictal aggression in epilepsy: Prevalence, clinical correlates, and psychosocial impact**

Isabelle Herion*, David Steinbart*, Desislava Dimova, Martin Holtkamp

* These authors contributed equally to this work.

**This file includes:**

**Table S1 to S4**

**Table S1: Overt aggression scale-modified (OAS-M)**

| **Type of aggression** | **Item** |
| --- | --- |
| **Verbal aggression** | Snapped or yelled at someone |
|  | Cursed or personally insulted someone |
|  | Engaged in a verbal argument with someone |
|  | Verbally threatened to hit someone subject knows |
|  | Verbally threatened to hit a stranger |
| **Aggression against objects** | Slammed door, kicked chair, threw clothes in anger |
|  | Broke something in anger |
|  | Broke several things in anger |
|  | Set fire, vandalized, or damaged another's property |
|  | Struck or injured/tortured a pet or other living  thing |
| **Aggression against others** | Made threatening gestures |
|  | Assault resulting in no physical harm to another |
|  | Assault resulting in some physical harm to another |
|  | Assault resulting in some physical injury to another |
|  | Assault that required medical attention |
| **Aggression against self** | Hit, bit, or scratched self |
|  | Banged head, or hit fists against wall |
|  | Cut, bruised, or burned self, but only superficially |
|  | Cut, bruised, or burned self deeply or seriously |
|  | Broke teeth, bone, or skull |

Items of Overt aggression scale-modified (OAS-M) as developed by Emil F. Coccaro (*Coccaro EF. The Overt Aggression Scale Modified (OAS-M) for clinical trials targeting impulsive aggression and intermittent explosive disorder: Validity, reliability, and correlates. J Psychiatr Res. May 2020;124:50-57. doi:10.1016/j.jpsychires.2020.01.007*), stratified by the sub-components. The manifestations of aggression are quantified on six levels from 0 (no events within that category) to 5 (most severe form of assault within that category). Additional weights are added for some of the sub-score of aggression: Aggression against objects is considered more severe than verbal assault and its score is multiplied by “2”; aggression against others and aggression against self are considered more severe than aggression against objects, and the respective score is multiplied by “3”.

**Table S2: Comparison of OAS-M and subjective occurrence of PIA based on self-reports and external reports**

| **Comparison of self-report (n=201)** | **OAS-M self ≥ 2**  (n=24, 12.0%) | **OAS-M self < 2**  (n=177, 88.0%) |
| --- | --- | --- |
| **PIA (subjective occurrence) self-report** (n=33, 16.4%) | 23 (95.8%) | 10 (5.6%) |
| **Comparison of external report (n=30)** | **OAS-M external ≥ 2** (n=5, 16.7%) | **OAS-M external < 2** (n=25, 83.3%) |
| **PIA (subjective occurrence) external report** (n=8, 26.7%) | 5 (100%) | 3 (12.0%) |

N: number of patients.

**Table S3: Number of self-reported subjective postictal aggression stratified by seizure type**

| **Seizure type**    **Number** | Bilateral tonic-clonic seizures/ Generalized tonic-clonic seizures  (n = 108) | Focal impaired consciousness seizures  (n = 73) | Focal preserved consciousness seizures  (n = 75) | Absence seizures  (n = 8) |
| --- | --- | --- | --- | --- |
| 1x/ year | 7 (6.5%) | 3 (4.1%) | 1 (1.3%) | 0 |
| 2-5x/ year | 6 (5.6%) | 1 (1.4%) | 3 (4.0%) | 0 |
| 6-8x/ year | 1 (0.9%) | 2 (2-7%) | 1 (1.3%) | 0 |
| > 8x/ year | 0 | 5 (6.8%) | 5 (6.7%) | 2 (25.0%) |
| Never | 94 (87.0%) | 62 (84.9%) | 65 (86.7%) | 6 (75.0%) |

Number of subjective occurrence of postictal aggression (PIA) after different seizure types. In total, 33 patients self-reported subjective occurrence of PIA. Multiple seizure types per patient possible.

**Table S4: Frequency of postictal aggression stratified by antiseizure medication**

| **Antiseizure medication (n (%))** | **Patients with PIA  (OAS-M ≥ 2)**  n = 24 | **Patients without PIA (OAS-M < 2)**  n = 177 |
| --- | --- | --- |
| Lamotrigine | 12 (50.0%) | 59 (33.3%) |
| Levetiracetam | 8 (33.3%) | 77 (43.5%) |
| Lacosamide | 4 (16.7%) | 38 (21.5%) |
| Valproate | 4 (16.7%) | 14 (7.9%) |
| Perampanel | 2 (8.3%) | 13 (7.3%) |
| Brivaracetam | 3 (12.5%) | 11 (6.2%) |
| Oxcarbazepine | 2 (8.3%) | 11 (6.2%) |
| Carbamazepine | 0 (0.0%) | 9 (5.1%) |
| Zonisamide | 0 (0.0%) | 8 (4.5%) |
| Eslicarbazepine | 0 (0.0%) | 7 (4.0%) |
| Topiramate | 1 (4.2%) | 4 (2.3%) |
| Primidone | 0 (0.0%) | 4 (2.3%) |
| Gabapentin | 1 (4.2%) | 2 (1.1%) |
| Pregabalin | 1 (4.2%) | 2 (1.1%) |
| Phenytoin | 0 (0.0%) | 1 (0.6%) |

Antiseizure medications in mono- or polytherapy, stratified by occurrence of postictal aggression, defined by Overt aggression scale modified (OAS-M) ≥ 2. N: number of patients.
